# Burn-associated metabolic dysfunction: could extracellular vesicles play a role?

**DOI:** 10.1042/CS20260147

**Published:** 2026-04-17

**Authors:** Mahmoud Farahat, Ghazaleh DadashiZadeh, Zachary Ricciuti, Alexandra Pappas, Marc G. Jeschke

**Affiliations:** 1 Department of Surgery, McMaster University, Hamilton, Ontario, Canada; 2 Center for Burn Research, Hamilton Health Sciences, Hamilton, Ontario, Canada; 3 David Braley Research Institute, Hamilton, Ontario, Canada; 4 Department of Medical Sciences, Faculty of Health Sciences, McMaster University, Hamilton, Ontario, Canada

**Keywords:** Adipose browning, Burn injuries, Extracellular vesicles, Hypermetabolism

## Abstract

Hypermetabolism is a well-recognized component of the detrimental systemic response following severe burn injury. Despite extensive clinical observations and research output, the underlying mechanisms that drive this persistent metabolic dysregulation remain poorly understood. One emerging area of interest is the role of extracellular vesicles (EVs), small membrane-bound particles released by cells that are increasingly recognized as important mediators of intercellular communication and contributors to various pathological conditions. Recent studies have highlighted their significant involvement in metabolic regulation, acting as carriers of bioactive molecules that influence metabolic pathways in recipient cells. EVs contribute to the modulation of glucose and lipid metabolism, insulin sensitivity, mitochondrial function, and inflammation, thereby influencing systemic metabolic homeostasis. In this context, EVs have been proposed as potential mediators in the metabolic problems that follow severe burn injuries. Burn-induced hypermetabolism, marked by insulin resistance, muscle wasting, and systemic inflammation, is affected by exosome-driven signaling between damaged tissues and metabolic organs. Exosomes released after a burn carry inflammatory cytokines, stress-response proteins, and metabolic regulators that change cellular functions in distant organs such as the liver, skeletal muscle, and adipose tissue. Understanding how exosomal communication contributes to post-burn metabolic issues could lead to new diagnostic and treatment options for improving recovery and metabolic health in burn patients. This review aims to clarify the role of EVs in burn-induced metabolic problems, emphasizing their potential as new mediators in the complex systemic response, which may provide new insights into the underlying mechanisms and help identify novel therapeutic targets.

## Introduction

Extracellular vesicles (EVs) are spherical phospholipid nanoparticles with diverse cargo components, secreted by virtually all cells, each specifically modifying the membrane protein and lipid composition to direct their vesicles toward their desired destination. Our expanding knowledge of EVs’ cargo has revealed that EVs carry key biological substrates—proteins, lipids, DNA, and RNA—that provide necessary information within intercellular and interorgan communication networks [1]. EVs first garnered attention in the 1960s when they were identified in bone cartilage; however, it was not until 1981 that Trams et al. provided evidence of their role in cellular communication [2]. In this pioneering work, they demonstrated that EVs from mammalian cell lines could dephosphorylate cell surface proteins, underscoring their potential to stimulate signaling pathways within these cells [3]. Building on this, accumulated findings established exosomes, the most prevailing EVs, as key players in numerous physiological processes such as immune regulation, neuron-glial communication, regulation of bone formation, and metabolism [4]. Moreover, persistent explorations of EVs have revealed their significant potential as therapeutic tools for a range of disorders, including cancers and Alzheimer’s disease. In the context of metabolism, EVs were shown to play a key role in mediating metabolic homeostasis physiologically and pathologically, offering a promise for their utilization as disease biomarkers and/or treatment options [5,6]. For the latter, the advent of EVs as a treatment for metabolic disorders, like diabetes, has substantially increased the interest, reflected by a growing body of research [7,8].

Severe burn injuries are often associated with marked metabolic disorders that manifest as a strong hypermetabolic response. This presents an imminent therapeutic challenge due to its enduring persistence far beyond the actual burn wound healing (up to 5 years in humans) [9,10]. Post-burn hypermetabolism induces a cascade of severe and prolonged responses, including hyperlipidemia, fatty liver disease, and adipose tissue (AT) browning [11]. The underlying mechanisms of burn-associated metabolic disorders, despite their impact, are essentially unknown. As such, effective therapeutics have fallen short of providing a solution for millions of burn patients. Interestingly, recent reports have shown a remarkable response in the multitude of circulating EVs, their cargo composition, with these changes correlating directly with the severity of the burn injury [12,13]. Additionally, EVs released after burns have been shown to elicit immune responses in sham-treated mice that mirror those observed in burn-injured animals [14]. This evidence, together with the EVs’ ability to carry a plethora of bioactive molecules, strongly suggests EVs as key messengers linking burn-induced cellular stress to the widespread metabolic dysregulations observed post-injury.

As evidence mounts for the therapeutic potential of EVs in metabolic disorders, it is essential to identify ways to translate these findings to burn-induced metabolic conditions. In this review, we will explore our current knowledge of EVs as mediators and therapeutic agents for metabolic diseases. Additionally, we will consider how these insights may apply to burn injuries, assessing their potential to tackle post-burn metabolic complications.

### EVs: nature, biogenesis, function

**Figure 1:**
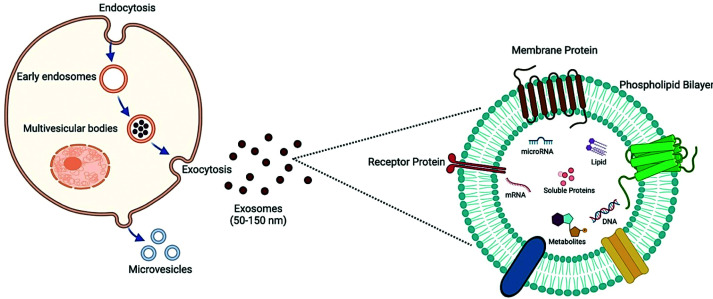
Exosomes structure and biogenesis Microvesicles are formed by direct budding from the plasma membrane, while exosomes originate from the inward budding (exocytosis) of the multivesicular body (MVB) membrane. Once released, exosomes interact with recipient cells via protein–protein or receptor-ligand binding, triggering signaling cascades and endocytosis. The biological effects of exosomes are determined by their cargo, which includes proteins, lipids, RNAs (miRNA and mRNA), and DNA fragments. *Created in BioRender. Jeschke, M. (2026)* https://BioRender.com/sioy668

EVs are unique biological devices of communication, formed within and released by cells, each carrying its unique set of bioactive cargo. EV can work locally by altering the function and phenotype of neighboring cells or traveling through the circulation to act on distant tissues. Structurally, EVs are lipid-enclosed sacs, classified by their size, biogenesis, and cargo into three main subpopulations: exosomes, microvesicles (MVs), and apoptotic bodies (ApoBDs). MVs and ApoBDs are the largest of the three, measuring at 100 nm–1 μm and 1–5 μm, respectively, and both share similar pathways of biogenesis, highlighted by the outward budding of the plasma membrane [15]. More specifically, the formation of these vesicles requires plasma membrane phospholipid redistribution, ROCK1-mediated modulation of the plasma membrane, and ARF6-mediated coordination of actomyosin contraction, which is necessary for fission [16,17]. Conversely, exosomes—the smallest of the EVs, sitting at a mere 20–100 nm—are synthesized from the endosomal membrane, a process mediated by the accumulation of various tetraspannins and requiring the activity of complexes referred to as “endosomal sorting complex required for transport” (ESCRT) [18,19]. EVs display extreme heterogeneity, which influences not only specific aspects of their biogenesis but also their uptake into and interaction with target cells (Figure 1). EVs’ cellular origin, size, expressed surface proteins, and other molecular factors determine their target cell destination and mode of entry [20]. Generally, EVs will enter a cell through fusion with the plasma membrane or via endocytotic pathways, which include clathrin-mediated endocytosis, caveolin-mediated endocytosis, lipid raft-mediated endocytosis, micropinocytosis, and phagocytosis [20,21]. The decision on which path the EV will travel through depends upon the characteristics of the EV itself and the type of recipient cell. For example, neurons accept EVs through clathrin-dependent endocytosis or phagocytosis, displaying a cell-specific mode of entry, whereas exosomes released by B cells will selectively bind to follicular dendritic cells, highlighting EV-dependent target cell selection [22–24]. HeLa cells and EBV-transformed B cell lines release clusters of exosomes, influencing target cells to favor phagocytosis or micropinocytosis to support the ingestion of large, aggregated EVs [23,25]. These examples highlight the delicate and complex transportation mechanism of EVs. Therefore, any deviation from the homeostatic balanced condition, such as disease and/or injury, would impact his mechanism and elicit changes in EV production, nature, and uptake.

Mainly, EVs exert their action by releasing their cargo into the recipient cell’s cytoplasm. However, to safely deliver their cargo, EVs must avoid their most dreaded fate, lysosomal degradation. An EV can directly fuse with the plasma membrane, entering the recipient cell and concurrently releasing its cargo into the cytoplasm. However, concomitantly, they become susceptible to lysosomal degradation. Some endocytosed EVs evade lysosomal degradation by fusing back with the limiting endosome membrane, allowing the release of their content into the cytoplasm, and maximizing the use of their cargo [26]. Interestingly, some EVs exert their effects without entering the cell by binding to and activating extracellular receptors on recipient cells. Indeed, it was demonstrated that B cell- and dendritic cell-derived exosomes induced specific T cell antigenic responses by antigen presentation [27,28].

EVs carry an assortment of cargo, including DNA, mRNA, miRNA, lipids, metabolites, and various proteins (Figure 1). Each EV’s cargo contains its own proportioned set of these bioactive molecules, conferring unique origin and functionality. Generally, EVs are highly abundant in cytoskeletal, cytosolic, heat shock, plasma membrane, and vesicular trafficking proteins [29]. If internalized via endocytosis, these proteins will either be degraded or appropriately processed for subsequent use within the cell. For instance, intestinal epithelial- or dendritic cell-derived exosomes’ cargo that is processed in the endocytic compartment of other dendritic cells and subsequently used for antigen presentation [30,31]. In contrast, EVs contain several forms of RNA [32], including ribosomal RNA, fragments of tRNA, Y-RNA, and vault-RNA [33–35]. All of which must either be released directly into the cytoplasm or fuse out of the endosome to elicit its functions [36,37]. Additionally, EVs can carry a diverse set of messenger RNAs (mRNAs). For example, human mesenchymal stem cell-derived EVs (MSCs-EVs) were reported to contain 239 mRNAs involved in key cellular processes such as proliferation, immune regulation, differentiation, and transcription [38]. Such stem-cell-derived EVs protected against glycerol- or cisplatin-induced kidney injury both *in vivo* and *in vitro* [38,39]. MicroRNAs (miRNAs) are another form of RNA with significant functional importance. EV-bound miRNAs have been implicated in various physiological processes such as muscle cell differentiation [40], follicular maturation [41], and immune system regulation [42]. For instance, EVs from different T-cell subsets, such as Th1, Th2, and Treg, carry varying miRNA signatures, with Treg cells’ exosomes reported to suppress pathogenic Th1 cells and prevent inflammation [43]. DNA cargo in EVs, on the other hand, is relatively unknown despite its physiological significance. The DNA contained within tumor cell-derived EVs reflects the mutational status of the malignant cells, offering a key insight into their genetic alterations and supporting the potential of EVs as cancer biomarkers [44–46]. Finally, beyond serving as a structural component in the EV membrane, EVs can carry various bioactive lipids, including eicosanoids, fatty acids, and cholesterol, highlighting their role in regulating bioactive lipid signaling [47]. EV-mediated lipid transfer has been linked to key physiological processes; for example, EV-derived lipids can induce cell death in pancreatic tumoral cells via Notch signaling activation [48]. Additionally, EV-delivered lysophosphatidylcholine promotes immune activation through dendritic cell maturation and lymphocyte chemotaxis [49].

### The hidden player—EVs as key regulators in metabolic regulation

As EVs were established as a main intercellular communication venue, their role as a central modulator of physiologic processes became more evident. Metabolism is a highly coordinated physiological process that tissues use to generate energy for various cellular functions. Being regulated by a host of endogenous and exogenous factors, interorgan communication among different metabolic organs proved to be the cornerstone to maintaining metabolic homeostasis [50]. Apart from the widely known factors such as hormones (e.g., insulin, glucagon), adipokines (e.g., adiponectin, leptin) [51], and fatty acids [52,53]. As summarized in Table 1, EVs from key metabolic organs such as the pancreas, skeletal muscles, AT, and liver have recently been recognized to play a significant role in metabolic homeostasis and pathological conditions in metabolic organs. Their detailed effects and modes of action are discussed in this section.

#### Pancreatic beta cells

The pancreas plays a central role in metabolic regulation, with pancreatic β-cells maintaining glucose homeostasis through insulin secretion. Several studies have demonstrated the regulatory role of exosomes in the development and fate of β-cells. Guay et al. suggested that miRNA-loaded exosomes act as a novel mechanism for regulating the activity of pancreatic beta-cells [54]. They have shown that MIN6B1 beta-cells can modify their exosome miRNA content in response to diabetes-associated pathophysiological stimuli and further transfer these miRNA-loaded exosomes to neighboring MIN6B1 cells. Interestingly, it has been shown that cytokine-treated MIN6B1 exosomes can induce apoptosis in recipient MIN6B1 cells [55]. Similarly, Lakhter et al. showed that cytokine (i.e., IL-1β, IFN-γ, and TNF-α) treatment of β-cell lines and human islets resulted in a significant enrichment of β-cell EV miR-21-5p without affecting the number of released exosomes [56]. Utilizing INS-1 rat pancreatic cells, Zhu and colleagues showed that concentrations of cytokine treatment inversely affect neutral ceramidase (NCDase) release in INS-1 cells’ exosome cargo. Furthermore, NCDase-containing exosomes isolated from low-concentration cytokine-treated INS-1 cells could inhibit apoptosis induced by a high concentration of cytokines via the production of sphingosine 1-phosphate (S1P) and its activation of S1P receptor 2 [57]. In addition to the intrinsic exosomes released from pancreatic cells, Jalabert et al. showed that miR-16 was overexpressed in skeletal muscle exosomes from mice fed a 20% palmitate-enriched standard diet. When taken up by MIN6B1 cells, these miR-16-rich exosomes regulated Ptch1, a gene involved in pancreas development, resulting in MIN6B1 proliferation in vitro and an increased pancreatic islet size *in vivo* [58] (Table 1).

#### Skeletal muscle

Skeletal muscle plays a major role in glucose homeostasis as a major insulin-dependent glucose uptake site. Moreover, the insulin-resistant state of skeletal muscles is considered a leading cause of type 2 diabetes development [59]. Recent research has shed light on skeletal muscle-derived exosomes as potent metabolic mediators, acting in both paracrine and endocrine manners. As for the former, muscle cell-released exosomes were shown to be transferred to neighboring muscle cells, affecting their cell cycles and differentiation [40]. Interestingly, muscle cells were shown to alter their exosome quantity and cargo when exposed to fatty acids. For instance, muscle-derived exosomes from obese and insulin-resistant, HP-fed mice (standard diet enriched with 20% palm oil) were shown to induce myoblast proliferation and alter the expression of genes involved in the cell cycle, adhesion, and muscle differentiation [60]. As for the endocrine nature, MIN6B1 pancreatic cells were shown to uptake skeletal muscle exosomes in vitro from mice fed either a standard chow diet (SCD) or an HP-fed diet. Interestingly, only the latter could induce the proliferation of MIN6B1 cells and isolated pancreatic islets. Furthermore, mice injected with labeled exosomes from muscle cells showed a preference for their accumulation in several metabolic tissues, such as the pancreas, liver, gastrointestinal tract (GIT), and distal muscles, suggesting that muscle exosomes play a potential role in the inter-organ network governing glucose homeostasis and metabolism [58,60] (Table 1).

#### Adipose tissue

Recent evidence has shown that adipose tissue (AT) can widely communicate with other organs and play a central role in regulating whole-body metabolism through the release of adipokines [61]. AT is a highly heterogeneous and dynamic organ that plays important roles in regulating energy metabolism and insulin sensitivity. In addition to its classical roles in nutrient sensing and energy storage/dissipation, AT secretes a large number of bioactive molecules termed adipokines, participating in immune responses and metabolic regulation through their paracrine and/or endocrine actions [62]. Adipose-derived extracellular vesicles (ADEVs) have recently emerged as a novel class of signal messengers, mediating intercellular communications and inter-organ cross-talk, and therefore, can contribute to the onset and progression of systemic metabolic dysfunctions such as type 2 diabetes and fatty liver disease. At the same time, because EVs can modulate gene expression and trigger targeted functional responses in recipient cells depending on their cargo, they also provide a mechanism for repairing metabolically impaired tissues. Moreover, the distinct cargo signatures carried by EVs in various metabolic conditions underscore their promise as biomarkers for detecting and monitoring metabolic dysfunction [63].

AT can adjust exosome production and release in response to changes in metabolic condition and/or metabolic dysfunction. For instance, obesity-associated metabolic syndrome was associated with a significant increase in circulating EVs containing adiponectin but not leptin [64]. Furthermore, adipocytes can enhance their exosome release in response to antilipolytic and lipogenic signals, such as excess fatty acids (e.g., palmitate) and reactive oxygen species (ROS) [65]. In the same context, Deng et al. have shown that the release of exosomes is enhanced in the AT of obese (*ob/ob*) and high-fat diet-fed mice compared with control lean mice [66]. Such response was not limited to the number of exosomes released but also to the exosome cargo content. For example, analysis of EVs isolated from the AT of morbidly obese donors revealed distinct protein expression profiles in visceral (VAT) versus subcutaneous (SAT) compartments. Notably, VAT-derived EVs were enriched in proteins linked to adipose inflammation and insulin resistance, including TGFBI, CAVN1, CD14, mimecan, and thrombospondin-1 [67]. In the same context, exosomes from obese mice AT were shown to have higher levels of the retinol-binding protein-4 (RBP-4) protein. RBP-4 can activate the TLR4/NFκB pathway, promoting macrophages’ release of IL-6 and TNF-α and inducing systemic insulin resistance [54]. Moreover, hypertrophic obese AT tends to release exosomes loaded with pro-inflammatory and insulin resistance-inducing factors [68]. Camino et al. showed that insulin-resistant and lipid-hypertrophied adipocytes shed EVs capable of inducing hypertrophy and/or insulin resistance in recipient healthy adipocytes. Moreover, they demonstrated that these EVs can enhance IL-6 and TNFα expression and induce macrophage inflammation [69]. Likewise, Eguchi and colleagues found that adipocytes from *ob/ob* mice release exosomes that can directly induce monocyte-to-macrophage differentiation [70]. Similar results were reported by Zhang et al., showing that adipocyte exosomes, highly enriched with miR-155 from high-fat diet-fed mice, induced the conversion of bone marrow-derived macrophages (BMDMs) toward an M1 profile [71]. Additionally, adipocytes secrete miR-34a-enriched exosomes, which are taken up by nearby macrophages, leading to the suppression of anti-inflammatory M2 polarization. Interestingly, deleting miR-34a in adipocytes protects mice from obesity-related metabolic decline [72]. Conversely, exosomes from M2-polarized macrophages (induced by IL-4) carry signals that promote browning and oxidative metabolism in adipocytes [73,74].

Concomitantly, exosomes released from adipocytes show different contents depending on the size and type of the releasing adipocytes. For instance, exosomes released from large adipocytes contain miR-16, miR-27a, miR-146b, and miR-222, which can be transferred to small adipocytes to induce lipogenesis and cell hypertrophy [75]. Additionally, exosomes released from brown adipocytes were shown to be transported to the liver through the circulation. Where they act to restore levels of several circulating miRNAs that are involved in glucose tolerance and the down-regulation of hepatic Fibroblast Growth Factor 21 (*Fgf21*) mRNA and circulating FGF21 levels [76]. Crewe et al. showed that adipocytes can secrete small EVs containing oxidatively damaged mitochondrial particles in response to mitochondrial stress. Such mitochondria-loaded EVs can enter circulation and are taken up by cardiomyocytes, which induces ROS production [77]. These accumulated results confirm the effect of metabolic conditions on the content of adipocyte-derived EVs and their potential endocrine effects by traveling through the circulation to distant tissues. AT is considered a major source of circulating exosome miRNAs, which play a crucial role in inter-organ metabolic communication. These adipose-derived miRNAs were shown to enhance cross-talk between metabolic organs [78]. For example, exosome miR-27a released from adipocytes can be taken up by skeletal muscle fibers, where it represses peroxisome proliferator-activated receptor gamma (PPARγ) expression and impairs insulin signaling [79].

Alongside adipocytes, stromal vascular fraction (SVF) represents a cellular component that harbors a heterogeneous cell population of resident immune cells, stem cells, fibroblasts, and endothelial cells [80]. Adipose tissue macrophages (ATMs), including classically activated proinflammatory M1-like or alternatively activated anti-inflammatory M2-like cells, constitute the largest immune cell population within the SVF [81]. During inflammatory conditions such as obesity, ATMs, especially M1, are steadily recruited to AT and proceed to release pro-inflammatory cytokines, including IL-6 and TNF-α [82]. Recent reports have shown that ATMs respond to inflammatory conditions by altering their exosome content. Ying and colleagues have demonstrated that exosomes from the ATMs (ATM-Exos) of either obese or lean mice can modulate insulin metabolism. Administration of obese mice’s secreted ATM-Exos to lean recipients resulted in glucose intolerance and insulin resistance. Conversely, hyperglycemic obese mice exhibited improved glucose tolerance and insulin sensitivity when receiving ATM-Exos from lean subjects [74]. Such a modulatory effect was further attributed to changes in the exosome miRNA content of both obese- and lean-derived ATMs [83,84] (Table 1).

#### Liver

Resembling the cellular structure of AT, the liver is composed mainly of parenchymal hepatocytes, which perform most of the liver function, alongside a variety of non-parenchymal cells, including hepatic stellate cells (HSC), sinusoidal endothelial cells (SEC), Kupffer cells (KC), liver-specific natural killer cells, and intrahepatic lymphocytes (IHL) [85]. EVs play a significant role in mediating intrahepatic cross-talk among these different cells, indicating their importance in regulating hepatic function in both health and disease [86,87]. Nojima and colleagues demonstrated that hepatocyte-derived exosomes can stimulate hepatocyte proliferation both *in vitro* and *in vivo*. Once internalized by the target hepatocytes, these exosomes transfer their cargo, rich in sphingosine kinase 2 (SK2), prompting the synthesis of sphingosine-1-phosphate (S1P) [88]. Conversely, EVs also play a crucial role in the development of common liver diseases such as non-alcoholic liver disease (NAFLD), non-alcoholic steatohepatitis (NASH), and alcoholic liver disease (ALD). Notably, damaged hepatocytes can secrete a substantial amount of EVs that contribute to inflammation and disease progression [89–91]. Furthermore, hepatocytes can modify their exosome cargo in response to various pathological conditions. For instance, in the early stages of obesity, hepatocytes release exosomes rich in miR-3075, showing a potent insulin-sensitizing effect in obese mice. However, during chronic obesity, hepatocyte exosomes are no longer enriched in miR-3075, resulting in the loss of their insulin-sensitizing effects, suggesting that hepatocytes adapt their exosome cargo as a physiological mechanism to combat insulin resistance in obesity [92].

On the other hand, the liver, as the main target for circulating EVs, primarily receives ADEVs that directly modulate hepatic metabolism. Based on their adipokine content, human ADEVs were shown to alter insulin signaling in hepatocytes *in vitro* [93]. Adiponectin-rich EVs from lean AT showed the capacity to induce insulin-sensitizing effects *in vitro* and to prevent liver and adipose inflammation when injected in high-fat diet-fed mice [94]. Conversely, EVs derived from obese AT can be taken up by hepatocytes *in vitro*, disrupting components of the transforming growth factor (TGF)-β signaling pathway, a mechanism that may contribute to the development of metabolic-associated liver disease (MASLD) [95] (Table 1).

**Table 1:** Extracellular vesicle (EV) sources, targets, and functional roles in metabolic regulation

EV cell source	Recipient organ or cell	Action	Effective cargo material	Reference
**Pancreatic Beta Cells**				
MIN6B1 beta-cells (mouse)	Neighboring MIN6B1 beta cells	Induce apoptosis	miR-29a, miR-146a	[55]
Islet cells (human)	Beta cells	Promote apoptosis	miR-21-5p	[56]
INS-1 cells (rat)	INS-1 beta cells	Inhibit cytokine-induced apoptosis via S1P receptor activation	NCDase	[57]
**Skeletal Muscle**				
Skeletal muscle cells (mouse)	MIN6B1 beta cells and pancreatic islets	Promote proliferation and increased islet size	miR-16	[58]
	Neighboring muscle cells	Induce myoblast proliferation; alter expression of cell cycle genes	miR-133, miR-206, miR-222, miR-223, miR-126	[40]
**Adipose Tissue**				
Obese adipocytes (mouse)	Monocytes	Induce insulin resistance via TLR4/NFκB pathway; induce differentiation to inflammatory macrophages	RBP-4	[54]
	Cardiomyocytes	Induce ROS production	Oxidatively damaged mitochondrial particles	[77]
	Skeletal muscle fibers	Repress PPARγ expression; impair insulin signalling	miR-27a	[79]
High-fat diet-fed adipocytes (mouse)	Bone marrow-derived macrophages	Induce M1 proinflammatory polarization	miR-155, miR-34a	[72,74]
Large adipocytes (rat)	Small adipocytes	Induce lipogenesis and cell hypertrophy	miR-16, miR-27a, miR-146b, and miR-222	[75]
Brown adipose tissue (mouse)	Liver	Restore glucose tolerance; down-regulate hepatic FGF21	miR-222	[76]
Adipose tissue macrophages (mouse)	Adipocytes	Modulate insulin metabolism	miR-29a	[84]
Lean adipose tissue (human)	Hepatocytes	Alter insulin sensitization	Adiponectin, IL6, MIF	[93]
**Liver**				
Hepatocytes (mouse)	Neighboring hepatocytes	Stimulate hepatocyte proliferation	SK2	[88]
	Metabolic tissue	Insulin-sensitization in early obesity	miR-3075	[92]

NCDase: neutral ceramidase; S1P: sphingosine 1-phosphate; RBP-4: retinol-binding protein-4; FGF21: Fibroblast growth factor 21; PPARγ: peroxisome proliferator-activating receptor γ; SK2: sphingosine kinase 2; MIF: macrophage migration factor.

### EVs in burn: mediators of response and repair

Burn injuries are a significant form of trauma that impose irreversible consequences, significantly impacting one’s health and quality of life. The WHO estimates that 11 million burn injuries occur annually worldwide, of which 180 000 are fatal [96]. Importantly, burn trauma does not occur uniformly, as both the depth and surface area influence outcomes and treatment methods. A severe burn is classified as one covering more than 10% of the total body surface area (TBSA) in older adults, 20% in adults, and 30% in children [97]. While the massive tissue destruction is widely perceived as the primary cause of high mortality and morbidity following a burn injury, the pathophysiological complications postburn are, in fact, the leading causes of significant postburn mortality and long-term morbidity [98]. In the wake of a burn injury, a range of systemic events is triggered corresponding to the severity of the injury and affects various systems. Hypermetabolism, sepsis, and muscle wasting are among the most detrimental pathophysiological responses induced by burns [99]. Understanding how these events initiate, mediate, and extend has been a focus of research for the past three decades. While accumulated research has unraveled a complex network of immune cells, cytokines, growth factors, hormones, and other molecules coordinating these responses, the full picture remains to be revealed [9]. The introduction of EVs as major mediators of cellular and interorgan communication strongly suggests they play a crucial role in postburn pathophysiological regulation. Despite recent advances in EV research in burn injuries, both in tissue repair and as biomarkers, the precise role of EVs in burn-associated metabolic disorders remains poorly understood. In the following section, we summarize key findings in burn EV studies, including therapeutic applications and links to metabolic changes. Next, we will outline post-burn metabolic alterations and compare them with well-characterized EV-mediated metabolic conditions. This comparative approach may help uncover how EVs contribute to the development and progression of post-burn metabolic disorders.

#### EVs in burn wound healing and hypermetabolism

##### EV-mediated tissue repair and inflammation resolution

Increasing evidence indicates that EVs actively promote tissue repair in burn injuries by enhancing cell survival, angiogenesis, and regeneration. For example, mesenchymal stem cell-derived exosomes (MSC-Exs) have been shown to accelerate wound closure and neovascularization in burn models [100]. In a rat model of deep second-degree burns, human umbilical cord MSC (UC-MSC) exosomes significantly improved wound healing, increasing angiogenic markers such as CD31 and promoting faster re-epithelialization [101]. Similarly, Mahdibpour and Ivari showed that human menstrual blood-derived mesenchymal stem cell EVs (MenSC-sEVs) could enhance neo-angiogenesis and accelerate wound closure in a third-degree burn mouse model [102]. Human MSC Exs (Hu-MSC-Exs) could mediate cutaneous regeneration by activating β-catenin and promoting skin stem cell proliferation during early repair, while suppressing Wnt signaling and cell expansion during the tissue remodeling phase [103]. They can carry anti-inflammatory signals that modulate the behavior of immune cells in burns. Notably, EVs derived from stem cells can deliver miRNAs that dampen inflammatory pathways. Li et al. demonstrated that exosomes from UC-MSCs rich in miR-181c suppress Toll-like receptor 4 (TLR4) signaling, thereby reducing macrophage secretion of TNF-α/IL-1β and alleviating the inflammatory response in a rat burn model [104]. These data showed the capabilities of exosomes in resolving inflammation, preventing excessive tissue damage, and further enhancing tissue regeneration, thereby preventing chronic inflammation or fibrosis. Collectively, these accumulated findings underscore the potential of the use of EVs in the context of burn wound healing [105]. On the other hand, the role of EVs in burn-induced metabolic dysfunctions and their potential use as a therapeutic strategy remains less explored than in wound healing, posing a gap in our burn care knowledge.

##### EVs as potential biomarkers of burn-induced hypermetabolism

Severe burns trigger a profound hypermetabolic state, which correlates with burn size and outcomes. Therefore, there is great interest in biomarkers that reflect this post-burn metabolic stress. Circulating EVs have emerged as promising diagnostic/prognostic biomarkers in this regard. Burn patients exhibit a significant surge in circulating EV numbers, accompanied by dynamic changes in EV cargo composition that correlate with injury severity and disease progression [106]. Importantly, recent human studies have begun to provide direct evidence linking burn-induced EV alterations with metabolic dysfunction. Longitudinal profiling of circulating EVs in burn patients has revealed time-dependent changes in EV size distribution, protein cargo, and inflammatory mediators that parallel the progression of hypermetabolism and systemic catabolism. For instance, temporal analyses demonstrated that EV-associated proteins involved in acute phase response, coagulation, and metabolic regulation are significantly enriched in patients with severe burns and prolonged hypermetabolic states [105,107]. While leukocytes and endothelial cells are identified as key sources of post-burn serum exosomes, circulating neutrophil-derived exosomes increase during the acute phase and then decline as the initial inflammation subsides. Functionally, burn EVs cause endothelial barrier disruption *in vitro*, leading to decreased trans-endothelial electrical resistance and gaps between cells [13]. Functionally, burn-derived EVs cause endothelial barrier disruption *in vitro*, contributing to vascular leak and edema formation. Beyond inflammatory signaling, emerging data suggest that EV cargo composition is closely associated with metabolic outcomes. Clinical studies have reported that EV-associated acute-phase proteins such as serum amyloid A1 (SAA1) and C-reactive protein (CRP) are significantly elevated in patients with severe burns and correlate with prolonged hospitalization, increased resting energy expenditure (REE), and worsened metabolic recovery [108]. These findings position EV cargo as a functional readout of systemic metabolic stress rather than merely a marker of inflammation. Moreover, proteomic analyses of human burn-derived EVs identified differential expression of proteins linked to key metabolic signaling pathways, including PI3K/AKT and insulin signaling [109]. Similarly, changes in EV-associated miRNAs in both human and murine models, such as miR-1, miR-133a, and miR-382-5p, have been implicated in the regulation of muscle metabolism, insulin sensitivity, and mitochondrial function [110].

Collectively, these findings highlight that burn-induced EVs are not only reflective of injury severity but are increasingly recognized as integrative biomarkers linking inflammation, metabolic dysregulation, and clinical outcomes in human burn patients. However, despite these advances, large-scale clinical validation studies remain limited, underscoring the need for more comprehensive and standardized investigations.

### EVs in post-burn metabolic disorders

#### Hypermetabolism

**Figure 2:**
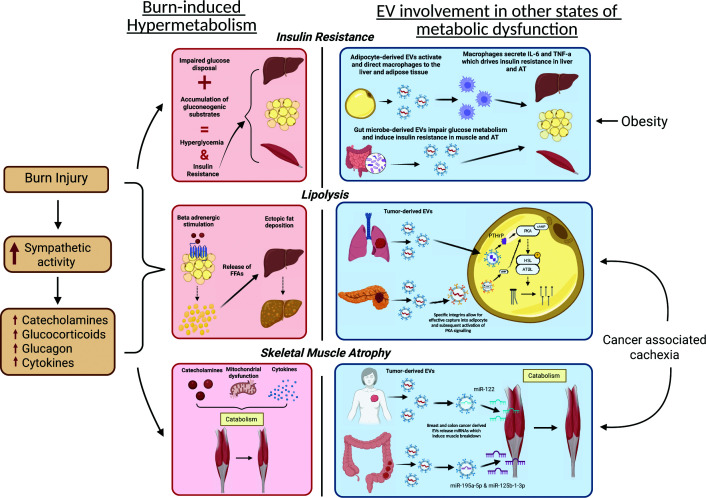
Post-burn hypermetabolism and EV-induced metabolic dysfunction: two manifestations of one process Burn injury triggers sympathetic activation and hormonal surges, leading to insulin resistance, increased lipolysis, and skeletal muscle atrophy—paralleling EV-driven mechanisms in other diseases where EVs modulate immune responses, lipolytic signaling, and muscle catabolism through targeted miRNAs and cytokines. *Created in BioRender. Jeschke, M. (2026)* https://BioRender.com/16we1o8

Typically, following a severe burn injury, an induction of a complex, catabolic, long-term stress response referred to as the hypermetabolic response ensues. Post-burn hypermetabolism is incomparable to similar trauma or disease-induced hypermetabolic conditions due to its magnitude and persistence, representing an unprecedented physiological response to trauma. Primarily, post-burn hypermetabolism is characterized by increased sympathetic activation and the release of stress hormones such as catecholamines, glucocorticoids, and glucagon, leading to heightened REE, peripheral insulin resistance, and the degradation of glycogen, proteins, and lipids [99]. This culminates in a deadly catabolic state associated with an increase in body temperature, oxygen, glucose utilization, CO_2_ production, and ultimately greater risk of organ failure, infection, and death. Although interventions such as the anabolic agent oxandrolone and the β-blocker propranolol demonstrate modest benefits, no therapy to date sufficiently attenuates the hypermetabolic response or prevents its long-term consequences. Therefore, new therapeutic avenues are urgent for modern burn care. The recent advances in EV discoveries offer a compelling new direction. Parallel EV-driven mechanisms have been identified in related metabolic and catabolic conditions, such as cancer cachexia and systemic inflammatory states, suggesting that EVs may act as key inter-organ regulators modulating metabolic dysfunction. As illustrated in Figure 2, these findings highlight a highly promising avenue, yet unexplored.

Insulin resistance is a particularly concerning complication following burn injury, as trauma-induced hyperglycemia has been strongly linked to impaired wound healing, skin graft loss, increased incidence of infection, and ultimately a greater risk of mortality [111]. Whether EVs are directly involved in burn-induced insulin resistance remains unknown; however, recent evidence from other metabolic conditions indicates that EVs play a role in the associated insulin resistance. For instance, murine models of obesity have shown that adipocyte-derived EVs can induce insulin resistance within the liver and ATs by transporting activated macrophages to the liver and AT, where they secrete TNF-α and IL-6, resulting in insulin resistance [112]. On the other hand, instead of relying on macrophage activation, adipocyte-derived EVs trigger insulin resistance in skeletal muscle via the release of exosome miR-27a, leading to reduced expression of insulin receptor substrate 1 (IRS-1) and glucose transporter GLUT4 [113]. Beyond AT, liver-derived EVs have also been shown to promote insulin resistance. Ji et al. reported that hepatic exosomes from high-fat diet obese mice carry pro-inflammatory signals that stimulate adipose macrophages, increasing tissue inflammation and thereby exacerbating systemic insulin resistance [92]. Like AT, lipid-stressed hepatocytes can exert paracrine effects by releasing saturated fatty acid (SFA)-enriched small extracellular vesicles (sEVs) [114]. SFA loaded sEVs are internalized by hepatic macrophages via TLR4, triggering a pro-inflammatory response that reduces insulin-stimulated AKT phosphorylation and impairs hepatocyte insulin signaling. Additionally, Choi et al. reported an increase in the production of gut microbe-derived EVs, which can induce insulin resistance and impair glucose metabolism within AT and skeletal muscle in high-fat diet mice [115] (Figure 2).

On the other hand, burn-induced hypermetabolism is associated with significant protein breakdown, which manifests in skeletal muscle atrophy and wasting that can persist for years following the injury [116]. While multiple mechanisms of action have been proposed, including cytokine-mediated catabolism, activation of proteolytic systems, mitochondrial dysfunction, insulin resistance, and immobilization, the full scope of mediators remains incompletely defined [117]. Investigations within other atrophy-inducing states have shown the involvement of EVs in cancer-associated cachexia (CAC), where they've been directly linked to the muscle wasting that occurs alongside breast and colon cancer [118,119]. Specifically, tumor-derived EVs can carry proteins and miRNAs that promote muscle wasting in preclinical models, suggesting a role for EVs as inter-organ catabolic signals [120]. Given the shared features between burn- and cancer-associated muscle atrophy, such as systemic inflammation and mitochondrial dysfunction, and WAT browning [121,122], it would be plausible that EVs might play a similar role in burn-associated muscle loss (Figure 2). Similarly, studies within CAC have found that cancer-derived EVs can induce lipolysis via fusing with adipocytes and triggering protein kinase A (PKA) signaling, as well as through membrane-bound integrins [123,124]. Interestingly, burn injuries elicit a strong lipolytic response fueled by increased β-adrenergic stimulation, leading to the elevation of circulating free fatty acids (FFAs) and a heightened risk of ectopic fat deposition [121]. Likewise, EVs may contribute to burn-induced lipolysis by propagating these catabolic signals, which intensify β-adrenergic-driven fat breakdown.

These findings underscore the potential for EVs to act as key mediators in the systemic metabolic dysregulation observed in burn patients. Future research should investigate whether similar EV populations, characterized by specific cellular origins and cargo, are up-regulated in response to burn-induced hypermetabolism.

#### Browning

AT comes in multiple forms with distinct functions. White adipose tissue (WAT) primarily stores energy, whereas brown adipose tissue (BAT) dissipates energy as heat. The “browning” of WAT refers to the process in which white adipocytes acquire characteristics of brown adipocytes, including increased mitochondrial content and up-regulation of thermogenic proteins like uncoupling protein 1 (UCP1). Known drivers for this phenomenon include exercise, cold exposure, hormonal factors, and pathological conditions, including cancer, cachexia, and severe burn injury [125].

##### Burn-induced browning

**Figure 3:**
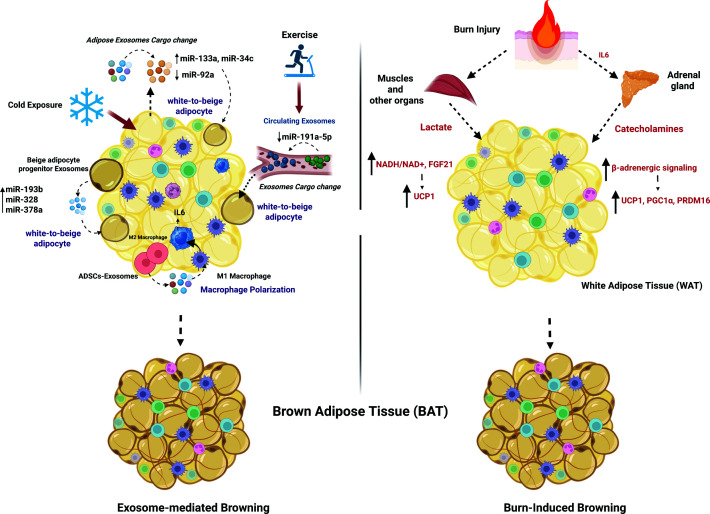
Mechanisms of burn- and EV-mediated WAT browning Schematic representation juxtaposing burn-induced systemic responses with EV-mediated signaling in promoting white-to-beige adipocyte conversion. Burn injury stimulates catecholamine release, activating β-adrenergic signaling pathways that drive adipocyte reprogramming. Concurrently, burn-associated metabolic stress and inflammatory mediators, including interleukin-6 (IL-6) and lactate, contribute to browning processes. Exosomes derived from BAT and adipose stem cells carry regulatory miRNAs, including miR-193b, miR-328, miR-378a, miR-92a, and miR-191a-5p, which promote thermogenic programming in recipient white adipocytes. Cold exposure and exercise further influence EV cargo composition, enhancing pro-browning signaling. In addition, EV-mediated cross-talk contributes to immune modulation, including macrophage polarization, which may further support AT remodeling. Collectively, these pathways converge to induce WAT browning and metabolic reprogramming following burn injury. *Created in BioRender. Jeschke, M. (2026)* https://BioRender.com/w4i9xpr

A hallmark of post-burn hypermetabolic response is the browning of WAT, characterized by the emergence of beige adipocytes within white fat depots and the up-regulation of UCP1, a key mediator of non-shivering thermogenesis [126]. This browning transformation enhances thermogenesis and energy expenditure, leading to the elevated REE observed in burn patients [127]. In the short term, browning serves as an adaptive healing mechanism; however, prolonged AT browning in burn patients may have deleterious effects. The increased metabolic demand can lead to lipolysis, fat mass depletion, insulin resistance, and impaired wound healing. Moreover, the chronic catabolic state places a significant burden on other metabolic organs, exacerbating systemic dysfunction [128]. Burn-induced browning is primarily driven by systemic inflammation and sustained catecholamine release from the adrenal gland, particularly norepinephrine, which activates β-adrenergic signaling pathways in adipocytes. This stimulation leads to increased cyclic AMP (cAMP) levels, activation of PKA, and subsequent up-regulation of key thermogenic genes UCP1, PGC1α, and PRDM16 [127]. Additionally, pro-inflammatory cytokines such as IL-6, which is markedly elevated in burn patients and originates from the bone marrow, have been shown to promote browning through both direct and indirect mechanisms, further amplifying energy expenditure. IL-6 appears to work in conjunction with recruited macrophages, promoting their transition to the M2 phenotype, which facilitates burn-induced browning. Mechanistically, macrophages recruited to burn-stressed subcutaneous white adipose tissue (sWAT) undergo alternative activation, leading to the expression of tyrosine hydroxylase and catecholamine production. These IL-6-mediated factors are crucial for inducing sWAT browning [129]. Additionally, Barayan et al. showed that burn-induced lactate increases act as a signaling mechanism to drive the browning of WAT in both burn patients and mouse models through increasing the intracellular redox pressure [NADH/NAD^+^] and enhancing expression of FGF21, proving that lactate shuttling plays a direct role in enhancing thermogenic gene expression and mitochondrial activity in AT post-burn [130] (Figure 3).

##### EV as a mediator of AT browning

Alongside classic hormonal signals (e.g., catecholamines), recent research highlights EVs, especially exosomes, as key mediators in intercellular communication that can influence adipose browning [131]. Exosomes can transfer bioactive cargo that triggers thermogenic programming in adipocytes or their precursors. A prominent mechanism is through miRNAs that alter gene expression in recipient cells. For instance, serum exosome miR-92a levels correlate inversely with BAT activity in humans, whereby healthy individuals with more active BAT had lower miR-92a [132]. Additionally, exosomes from beige adipocyte progenitors (adipose-derived stem cells undergoing browning) are enriched in miR-193b, miR-328, and miR-378a, which drive white-to-beige adipocyte reprogramming [133–135]. These miRNAs target pathways that promote mitochondrial biogenesis and uncoupling; therefore, treating obese mice with such exosomes attenuated weight gain and improved glucose homeostasis via enhanced WAT browning. Concomitantly, exosomes released by brown and beige adipocytes carry factors that can convert WAT to a brown-like state. In a pivotal animal study, researchers isolated exosomes from BAT and administered them to obese mice. The BAT-derived exosomes (i.e., BAT EVs) targeted white fat depots, inducing UCP1 expression and the development of beige adipocytes. Treated mice exhibited increased energy expenditure, reduced adiposity, improved glucose tolerance, and less fatty liver, indicating systemic metabolic benefits from WAT browning [136]. Moreover, a comparative analysis of BAT EVs and WAT EVs revealed that 60% of proteins were unique to BAT EVs. Strikingly, BAT cargo corresponded directly to the metabolic status, with BAT EVs from lean and obese rats showing differential protein expression [137]. Indeed, human brown adipocytes in culture secrete exosomes, and their output increases upon stimulation; such exosomes may represent an endocrine signal of thermogenic activation [113]. Meanwhile, adipose-derived stem cells (ADSCs) also release exosomes with browning potential during differentiation, which can directly induce browning in adipocytes, as shown by Jung et al. [138], and indirectly via macrophage polarization, as reported by Zhao et al. [139]. These reports underscore that cells within the adipose lineage can communicate browning signals via exosomes, which can reprogram white fat physiologically.

On the other hand, the mechanisms driving WAT browning continue to evolve, with emerging evidence linking EVs to traditionally recognized stimuli such as exercise, cold exposure, and cancer, as depicted in Figure 3. Although not yet fully elucidated, post-burn browning may likely be mediated through a similar action of EVs. Therefore, it is essential to examine EV-mediated WAT browning in other conditions and relate these insights to the context of burn injury.

Cold exposure is a classical trigger of BAT activation and WAT browning. Recent evidence suggests that cold not only activates nerves and hormones but also induces specific exosome communications. In humans, acute cold exposure has been shown to increase the release of BAT-derived exosomes (i.e., batosomes) into circulation, as reflected by elevated plasma levels of batosome proteins such as methylene tetrahydrofolate dehydrogenase (NADP^+^ dependent) 1-like (MTHFD1L), an exosome-mitochondrial enzyme. This indicates that browning signals can influence exosome biogenesis and content in activated thermogenic adipocytes by selectively loading exosomes with specific proteins and secreting them into circulation [140]. Chen et al. showed that cold-exposed mice exhibited major changes in adipose exosome content, including miR-92a, miR-133a, and miR-34c. Among these, miR-92a was consistently found to be reduced in BAT and WAT exosomes during cold exposure [132]. Interestingly, miR-92a has a known brown-inhibitory effect [141]. Therefore, this significant cold-induced miR-92a down-regulation could relieve brakes on adipose browning and possibly create a loop effect of browning/exosome reciprocal induction. Cold may also provoke other organs to send pro-thermogenic exosomes. Gao et al. showed that the liver senses cold and secretes exosomes enriched in miR-293-5p, which travel to brown fat and enhance its thermogenic gene expression (a liver-BAT axis in cold adaptation) [142] (Figure 3). Collectively, cold exposure can mediate the thermogenic browning response by controlling the exosome signaling (amount and content) both from and toward adipose depots.

Exercise is another potent modulator of adipose browning. Exercise is well known to improve metabolic health, and some of its benefits may be mediated by exerkines—exercise-induced signaling factors—including exosomes [143]. Long-term exercise has been shown to alter the miRNA cargo of circulating exosomes in a manner that promotes AT browning. In mice, plasma exosomal miR-191a-5p is significantly down-regulated following chronic exercise. Notably, miR-191a-5p normally targets the 3′UTR of *Prdm16* (a master regulator of beige fat development). Thus, exercise reduces levels of an anti-browning miRNA in exosomes, thereby relieving the suppression of PRDM16 and favoring the browning process. Consistently, exosomes from exercised mice promoted beige adipocyte formation when added to white adipocyte cultures, whereas exosomes from sedentary controls did not elicit this effect [144]. In obese mouse models, exercise training stimulated greater subcutaneous WAT browning compared with sedentary controls, and these changes were associated with altered circulating exosome miRNA profiles [79].

Interestingly, cancer cells were shown to release miRNA-enriched exosomes that actively induce pathological WAT browning, contributing to the development of cancer-associated cachexia. For instance, exosomes derived from breast cancer cells enriched with miR-204-5p are taken up by white adipocytes, where they suppress VHL expression, leading to stabilization of HIF1α and hyperactivation of leptin signaling. This cascade enhances lipolysis and initiates a robust browning program, ultimately promoting AT wasting [118]. Similarly, colorectal cancer-secreted exosome miR-146b-5p inhibits its target HOXC10, a negative regulator of brown fat gene programs, thereby up-regulating PRDM16 and driving white fat to a beige state [145]. Lung cancer exosomes offer yet another example: exosome miR-425-3p from tumor cells down-regulates adipocyte PDE4B, elevating cAMP levels and PKA activity. This leads to increased expression of UCP1, enhanced lipolysis, and induction of beige fat characteristics. *In vitro*, these tumor exosomes induce cultured 3T3-L1 adipocytes to acquire brown fat characteristics and release FFAs, while *in vivo*, they provoke loss of white fat and the appearance of UCP1-positive multilocular adipocytes [146,147].

Collectively, these findings highlight the pivotal role of exosomes in regulating AT browning. Acting as vehicles of inter-tissue communication, exosomes carry a variety of browning-inducing factors, including miRNAs (e.g., miR-193b, miR-328, miR-378a, and miR-425-3p) and proteins (e.g., STAT3 and PTHrP), which activate thermogenic gene programs in white adipocytes (Figure 3).

#### Burn-induced immunometabolic alterations

In AT, resident immune cells constitute the second most abundant cell type after adipocytes. These immune cells are essential for maintaining AT homeostasis, representing a wide range of immune cell types, including those from the myeloid lineage (macrophages, dendritic cells (DCs), mast cells, and granulocytes such as neutrophils, eosinophils, basophils) and the lymphoid lineage (T and B lymphocytes, natural killer (NK) cells, and natural killer T (NKT) cells [148]. The internal cross-talk between adipocytes and the diverse array of cells in the SVF, including fibroblasts, endothelial cells, and both innate and adaptive immune cells, is a major driver of AT inflammation [149,150]. For instance, during the post-burn hypermetabolic condition, immune cells accumulate excessively within the AT, fostering a pro-inflammatory environment. The infiltration of innate immune cells, such as neutrophils and macrophages, into injured tissue represents a pivotal event in initiating and orchestrating the complex processes required for effective tissue repair and regeneration [151].

##### EVs as key regulators of AT-immune responses post-burn

**Figure 4:**
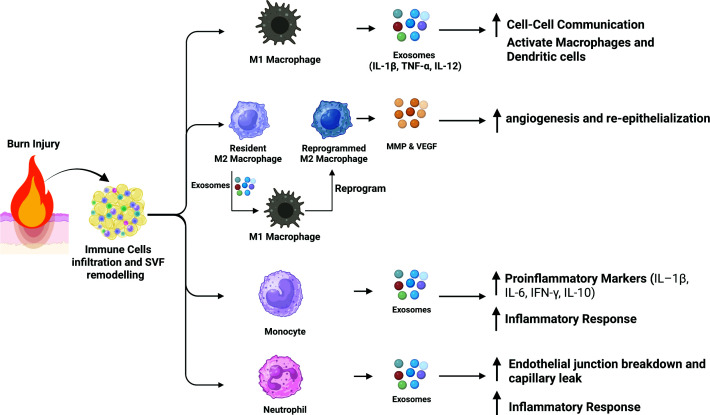
Exosome-mediated immune modulation and inflammatory responses after burn injury Burn injury causes immune cell infiltration and remodeling of the stromal vascular fraction (SVF). It prompts the release of proinflammatory mediators and EVs that enable communication between immune and stromal cells. These EVs carry inflammatory cytokines, such as interleukin-1β (IL-1β) and interleukin-6 (IL-6), which support the recruitment and activation of circulating monocytes and macrophages. Macrophage polarization and immune cell infiltration into AT and the SVF drive local and systemic inflammatory responses. EV-mediated signaling also contributes to endothelial dysfunction, resulting in the disruption of endothelial junctions and increased capillary permeability. These processes sustain inflammation and lead to immunometabolic dysregulation following burn injury. *Created in BioRender. Jeschke, M. (2026)* https://BioRender.com/i0v883d

Macrophages, the most abundant immune cell population in AT, comprise approximately 5% of SVF cells in rodents and 4% in humans [71]. In hypermetabolic conditions, such as those caused by burn injuries, macrophages show increased infiltration and mainly adopt a type 2 phenotype (alternatively activated M2 macrophages). During wound healing, exosomes from M2 macrophages (M2-Exo) can reprogram M1 macrophages into M2 macrophages with nearly 100% efficiency. The reprogrammed M2 macrophages (RM2) regain functionality by producing matrix metalloproteinases (MMPs) and vascular endothelial growth factor (VEGF), which are involved in healing processes such as angiogenesis and re-epithelialization [74]. Macrophage exosomes also facilitate cell–cell communication within the immune network. For instance, M1-EVs contain pro-inflammatory cytokines (IL-1β, TNF-α, IL-12) and miRNAs that can activate neighboring macrophages and dendritic cells further. In relation to burn injuries, burn-derived exosomes have been shown to alter macrophage gene expression. Exosomes from different post-burn phases modulated macrophage cytokine profiles according to the immune dysfunction stages, with exosomes at day 14 impairing macrophage phagocytosis [152]. Specifically, early-phase exosomes trigger a broad proinflammatory gene program in macrophages, including up-regulation of Myd88, NF-κB, IL-6, and IL-12 [75]. Such evidence suggests a role of exosomes in regulating adipose macrophage post-burn activity, including a possible induction of M1–M2 conversion and up-regulation of IL6 levels. Both could be linked directly to the burn-induced AT hypermetabolism. Reports also showed that plasma exosomes released following a burn injury can induce a unique set of immune genes in macrophages. Willis and colleagues found that burn-derived exosomes significantly up-regulated key immune genes, including MCP-1 (∼17-fold), IL-6 (∼3.3-fold), MyD88 (∼24-fold), and NFκB1 (∼150-fold), while sham exosomes down-regulated IL-6 with minimal effects on other genes (Figure 4). Interestingly, MCP-1, a chemokine secreted by adipocytes and stromal cells in AT in response to stress signals (e.g., nutrient excess, hypoxia, or inflammatory stimuli). MCP-1 signals through the C–C chemokine receptor 2 (CCR2) on circulating monocytes. The binding of MCP-1 to CCR2 triggers intracellular G-protein signaling, promoting chemotaxis and adhesion of monocytes to the endothelium, and driving their infiltration into AT [153]. Increased MCP-1 levels, such as those reported by burn-derived exosomes, can initiate a feed-forward loop in which recruited monocytes differentiate into macrophages and further secrete pro-inflammatory cytokines (such as TNF-α, IL-6, IL-1β) along with more MCP-1, thereby amplifying adipose inflammation and dysfunction [154].

Similar to macrophages, monocytes are activated very early after burn injury and release exosomes that disseminate inflammatory mediators systemically. Burn plasma exosomes are particularly enriched in monocyte/macrophage-derived factors. Strikingly, high-mobility group protein 1 (HMGB1) (a nuclear DAMP actively secreted by macrophages under stress) and IL-1β (a classic inflammasome cytokine) are co-packaged within EVs after burn [155]. These EVs often contain the pro-IL-1β form bound to HMGB1; notably, HMGB1 can chaperone pro-IL-1β and render it biologically active. When such exosomes interact with target cells, the HMGB1-IL-1β complex simultaneously engages IL-1 receptors and TLR4 on macrophages and other cells, triggering a synergistic inflammatory response. Indeed, EV-associated HMGB1/IL-1β heterocomplexes potently induce downstream cytokines (e.g., IL-6, IFN-β) in human macrophage-like cells, far more than either molecule alone [155]. This synergy helps drive the “cytokine storm” in acute burn injury. *In vivo*, transfusion of early burn exosomes into naive mice elevated serum IL-6, MCP-1, IL-8, IFN-γ, and IL-10, closely mirroring the cytokine profile of actual burn patients. The fact that exosomes alone can recapitulate these systemic effects underscores that macrophage-derived exosomes are central orchestrators of post-burn systemic inflammation (Figure 4).

IL-6, on the other hand, is considered an inflammatory adipokine that acts in an autocrine, paracrine, or endocrine manner. Chronic exposure of adipocytes to IL-6 has been shown to reduce insulin-stimulated glucose uptake, partly by up-regulating SOCS3, which interferes with insulin receptor substrates. Overexpression of IL-6 or persistently high IL-6 levels can promote AT inflammation, hepatic acute-phase responses, and insulin resistance. Collectively, it can be hypothesized that the up-regulation of MCP-1 and IL-6, induced by burn-derived exosomes, may play a cooperative role in driving post-burn hypermetabolism and AT browning. Specifically, MCP-1-mediated immune cell recruitment might initiate a localized inflammatory environment that primes AT for thermogenic remodeling, while IL-6 may act as a downstream effector that promotes progenitor cell differentiation into beige adipocytes and increases energy expenditure (Figure 4).

##### Current limitations in burn-EVs research

Despite the promising therapeutic and biomarker potential of EVs in burn care, several limitations constrain current progress. As discussed earlier, most available data validating the efficacy or prognostic value of EVs are in the context of wound healing rather than as metabolic interventions. These studies are preclinical, primarily in small animal (murine) models, with one human phase 1/2 clinical trial registered in patients receiving MSCs-Ex [156]. Additionally, unpublished data from our group demonstrated the efficacy of human-derived EVs in a large animal (porcine) burn model. This shortage of clinical or near-translational results reflects a broader pattern in EV-based therapies, where this wealth of preclinical data has not yet advanced to substantial translational progress. Such observation could be attributed to several issues, including the lack of standardized methods for EV isolation, characterization, and quantification. EVs are heterogeneous, varying in size, cargo composition, and cellular origin, which complicates reproducibility across studies [157]. Current isolation techniques, including ultracentrifugation, size-exclusion chromatography, and precipitation, differ significantly in EVs’ yield, purity, and scalability, leading to major inconsistencies in their applications [158]. Moreover, variability in characterization methods and the absence of generally accepted identification standards further complicate cross-study comparisons and result validation [159]. The optimal dosing is another key limitation in EV therapy. EV dosing across preclinical and clinical studies is reported using different metrics, including protein concentration, particle number, or originating cell count, making it difficult to establish an accurate dose-response relationship or compare therapeutic efficacy among studies [160]. Importantly, the method of EVs’ delivery to the targeted site or tissue is another major concern. EV distribution and therapeutic efficacy are highly dependent on the delivery route (e.g., intravenous, topical, inhalational), with each route presenting unique pharmacokinetic and targeting challenges. For instance, systemic delivery may result in rapid clearance or off-target accumulation, while localized delivery strategies may improve tissue specificity but face limitations in penetration and scalability [161]. These technical challenges, together with the complex post-burn environment characterized by hypermetabolism, persistent inflammation, immune dysregulation, and multi-organ involvement, further complicate the therapeutic application of EVs in burn injuries. This harsh environment can significantly alter EV stability, biodistribution, and cellular uptake, thereby limiting their therapeutic efficacy. For instance, increased vascular permeability and altered hemodynamics following burn injury may lead to unpredictable EV distribution and rapid clearance from the circulation [162]. Moreover, post-burn hyperinflammatory state and elevated levels of circulating proteases and ROS may compromise EV integrity and cargo stability, reducing their functional potency [163,164]. Additionally, burn-associated metabolic dysregulations, including insulin resistance, enhanced lipolysis, and mitochondrial dysfunction, may also influence the responsiveness of target tissues to EV-mediated signaling, thereby attenuating therapeutic outcomes. Additionally, burn-induced alterations in the extracellular matrix (ECM) can impair EV penetration and retention within target sites [98,165]. Finally, the progression of burn pathology, from acute inflammation to prolonged remodeling, requires precise control over EV dosing and timing of administration, which remains poorly defined in current translational studies.

In summary, advancing EV applications in burn-wound healing and hypermetabolism requires more comprehensive clinical trials, improved engineering and delivery methods, validated biomarkers in large patient cohorts, and clear guidelines for producing clinical-grade EVs. The following section outlines future steps to address these gaps, which will be crucial for translating EV-based diagnostics and therapies into effective burn care tools.

### Advancing EV research in burn injury: mechanistic insights and therapeutic potential

**Figure 5:**
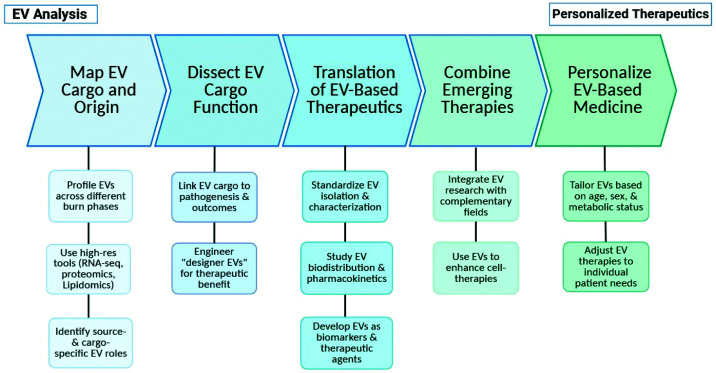
Overview of key research priorities for advancing EV research in burn injury Focus areas include EV mapping across burn phases, functional cargo analysis, therapeutic translation and clinical application, integration with regenerative platforms, and personalized medicine considerations. *Created in BioRender. Jeschke, M. (2026)* https://BioRender.com/12i989k

Building on the emerging role of EVs in regulating metabolic and immune responses following burn injury, future research should prioritize several critical aspects to expand both mechanistic understanding and translational potential (Figure 5).

#### Comprehensive understanding of EV origin and cargo across burn phases

A fundamental knowledge gap remains in our understanding of the temporal and spatial dynamics of EV release in response to burn injury. High-resolution profiling technologies, such as single-EV RNA sequencing, mass spectrometry-based proteomics, and lipidomics, should be employed to map the cellular sources and molecular cargo of EVs during acute (0–3 days), subacute (3–14 days), and chronic (>14 days) phases of burn trauma. Such granular analysis can identify distinct EV subtypes and their context-specific roles—whether deleterious (pro-inflammatory, pro-cachectic) or protective (anti-inflammatory, pro-regenerative). Special emphasis should be placed on delineating EVs derived from immune cells (e.g., M1 versus M2 macrophages, neutrophils, dendritic cells), mesenchymal/adipose progenitor cells, endothelial cells, and thermogenically activated or stressed adipocytes. This accurate mapping will serve as the base for the development of precision-targeted EV-based therapies.

#### Functional dissection of EV cargo in pathogenesis and recovery

A thorough functional analysis is required to establish the roles of EV cargo in driving or mitigating burn-induced pathophysiology. Murine models employing conditional knockouts, EV-specific reporter systems, or tissue-specific depletion of EV biogenesis pathways (e.g., Rab27a or nSMase2 deletion) can help elucidate the contribution of specific miRNAs (e.g., miR-193b, miR-328, miR-378a, miR-92a), proteins (e.g., STAT3, HMGB1, HSP70), and lipids to downstream effects such as AT browning, hepatic steatosis, muscle wasting, and systemic inflammation. In parallel, bioengineering of EVs—via *ex vivo* cargo enrichment or by reprogramming donor cells—could enable the creation of “designer EVs” with enhanced anti-inflammatory or metabolic reprogramming properties. For example, EVs from M2 macrophages or beige adipocyte progenitors enriched with browning-promoting miRNAs may serve as therapeutic tools to reverse burn-induced insulin resistance, adipose dysfunction, and chronic catabolism.

#### Translation and clinical development of EV-based therapeutics

Bridging the bench-to-bedside gap requires significant advances in EV standardization and clinical validation. Robust and scalable protocols for EV isolation (e.g., size exclusion chromatography, ultrafiltration, immunoaffinity capture), characterization (e.g., nanoparticle tracking analysis, flow cytometry, electron microscopy), and cargo quantification must be standardized to ensure reproducibility across laboratories. Additionally, pharmacokinetic studies to determine biodistribution, half-life, and organ tropism of EVs—particularly when administered intravenously, intradermally, or via biomaterial scaffolds—are essential for optimizing therapeutic efficacy and safety. Dose escalation studies in preclinical burn models will help define therapeutic windows. Concurrently, circulating EVs should be explored as biomarkers for burn severity, metabolic status (e.g., REE and insulin sensitivity), and response to therapy. EV-based liquid biopsies could allow for minimally invasive patient monitoring and early intervention.

#### Synergizing EV-based therapies with emerging regenerative platforms

To maximize therapeutic impact, EV research should be integrated with complementary fields such as adipose progenitor cell therapy, immunometabolism, biomaterials, and tissue engineering. For instance, combining EV delivery with bioengineered scaffolds or hydrogels could provide spatially controlled release to injured or metabolically stressed tissues. Similarly, co-administration of EVs with pharmacologic agents (e.g., β3-adrenergic agonists, anti-inflammatory biologics) may enhance browning and regeneration while suppressing systemic inflammation. EVs may also be used to prime autologous cell-based therapies by enhancing their survival, integration, and function post-transplantation.

#### Personalized medicine, age, and sex-specific considerations

Burn patients exhibit substantial heterogeneity in immune response, metabolic resilience, and long-term outcomes, which are strongly influenced by both age and sex [166]. While these factors are increasingly recognized in clinical burn care, their implications in EV biology remain underexplored. Importantly, accumulating evidence indicates that both age and sex significantly influence post-burn metabolic responses, including hypermetabolism, insulin resistance, and muscle wasting. Clinical and experimental studies have shown that elderly patients exhibit impaired metabolic adaptation, prolonged catabolism, and reduced regenerative capacity, whereas sex-based differences, driven in part by hormonal regulation, modulate immune responses, AT function, and overall metabolic outcomes following burn injury [166].

Given that EVs are key mediators of intercellular communication and systemic metabolic regulation, EV cargo composition and function are likely also influenced by age- and sex-dependent biological differences [12,108]. For example, variations in AT distribution, macrophage polarization, and hormonal signaling (e.g., estrogen and androgen pathways) may alter the molecular cargo of EVs, including miRNAs, inflammatory mediators, and metabolic regulators, thereby shaping their downstream effects on target tissues such as liver, muscle, and AT [167].

In the context of burn-induced hypermetabolism, such differences may result in distinct EV-mediated signaling profiles that contribute to variability in systemic energy expenditure, insulin sensitivity, and tissue remodeling between male and female patients, as well as between younger and older individuals [13]. Notably, recent studies demonstrate that EV-associated inflammatory signatures and cargo profiles correlate with burn severity and clinical outcomes, including sepsis and prolonged hospitalization, while the heterogeneous composition and cellular origin of EVs suggest context-dependent functional effects that may contribute to divergent patient responses [168].

Adjusting EV-based diagnostics and therapeutic strategies based on patient-specific variables, including age, sex, pre-existing metabolic status, and burn severity, may therefore enhance precision medicine approaches and improve clinical outcomes. Future studies should prioritize stratified analyses of EV cargo and function across age groups and between sexes, incorporating longitudinal human datasets and multi-omics approaches to better define how these variables influence EV-mediated immunometabolic regulation. Additionally, understanding pediatric versus adult EV responses is critical, given the distinct developmental and metabolic profiles that may uniquely shape EV biology and therapeutic responsiveness.

## Conclusion

Advancing EV-based research in the context of burn injury metabolic disorder offers a transformative opportunity to uncover novel pathophysiological mechanisms and develop next-generation, cell-free therapeutics. Through integrative approaches spanning high-resolution molecular profiling, functional validation, bioengineering, and clinical translation, EVs may emerge as both biomarkers and active agents in the fight against the long-term immunometabolic consequences of severe burns. Strategic investment in this interdisciplinary field promises to reshape burn care and improve outcomes for patients facing the complex systemic challenges that follow thermal trauma.

## Data Availability

All data were available in the manuscript. Additional data are not applicable.

## Open Access

Open access for this article was enabled by the participation of McMaster University in an all-inclusive *Read & Publish* agreement with Portland Press and the Biochemical Society under an agreement with OCUL.

## Abbreviations

ADEVs: Adipose-derived extracellular vesicles
ALD: Alcoholic liver disease
ApoBDs: Apoptotic bodies
AT: Adipose tissue
BAT: Brown adipose tissue
BMDMs: Bone marrow-derived macrophages
CAC: Cancer-associated cachexia
CM: Conditioned medium
EVs: Extracellular vesicles
FFAs: Free fatty acids
Fgf21: Fibroblast Growth Factor 21
IHL: Intrahepatic lymphocytes
IL-6: Interleukin-6
KC: Kupffer cells
MVs: Microvesicles
NAFLD: Non-alcoholic fatty liver disease
NASH: Non-alcoholic steatohepatitis
RBP-4: Retinol-binding protein-4
REE: Resting energy expenditure
S1P: Sphingosine-1-phosphate
SEC: Sinusoidal endothelial cells
SVF: Stromal vascular fraction
TBSA: Total body surface area
UCP1: Uncoupling protein 1
WAT: White adipose tissue
